# Discovery of Stress Responsive DNA Regulatory Motifs in Arabidopsis

**DOI:** 10.1371/journal.pone.0043198

**Published:** 2012-08-13

**Authors:** Shisong Ma, Shawn Bachan, Matthew Porto, Hans J. Bohnert, Michael Snyder, Savithramma P. Dinesh-Kumar

**Affiliations:** 1 Department of Plant Biology and the Genome Center, College of Biological Sciences, University of California Davis, Davis, California, United States of America; 2 Departements of Plant Biology and Crop Sciences, University of Illinois at Urbana-Champaign, Urbana, Illinois, United States of America; 3 Department of Genetics, Stanford University, Stanford, California, United States of America; Beijing Institute of Microbiology and Epidemiology, China

## Abstract

The discovery of DNA regulatory motifs in the sequenced genomes using computational methods remains challenging. Here, we present MotifIndexer - a comprehensive strategy for *de novo* identification of DNA regulatory motifs at a genome level. Using word-counting methods, we indexed the existence of every 8-mer oligo composed of bases A, C, G, T, r, y, s, w, m, k, n or 12-mer oligo composed of A, C, G, T, n, in the promoters of all predicted genes of *Arabidopsis thaliana* genome and of selected stress-induced co-expressed genes. From this analysis, we identified number of over-represented motifs. Among these, major critical motifs were identified using a position filter. We used a model based on uniform distribution and the z-scores derived from this model to describe position bias. Interestingly, many motifs showed position bias towards the transcription start site. We extended this model to show biased distribution of motifs in the genomes of both *A. thaliana* and rice. We also used MotifIndexer to identify conserved motifs in co-expressed gene groups from two Arabidopsis species, *A. thaliana* and *A. lyrata*. This new comparative genomics method does not depend on alignments of homologous gene promoter sequences.

## Introduction

The mechanisms behind regulated gene expression have been subject of passionate discussions for generations of molecular biologists. Though the list for such mechanisms has constantly been broadened, promoter motif based regulation remains an extensively studied topic of importance for eukaryotic gene expression. DNA regulatory motifs, or cis-regulatory elements, are 5–15 base pair (bp) long nucleotides within the promoter region that function primarily as transcription factor (TF) binding sites, although binding for other proteins, such as kinases, has also been reported [1]. The interaction between TFs and their target motifs can lead to induction or repression of gene expression. Various methods have been used to identify motifs, including deletion based functional analysis, comparative genomics, analysis of co-expressed genes, and ChIP-chip or ChIP-seq [2], [3], [4].

It is assumed that co-expressed genes are likely to contain a set of over-represented motifs in their promoters that lead to their similar expression patterns. Various computational methods have been developed to discover such motifs. These methods can be divided into two categories based on their ways to represent motifs: those using a probabilistic sequence model and those using word-based methods [3], [5]. Probabilistic approaches describe motifs as position weight matrix (PWM), where parameters are optimized using maximum-likelihood principles or Bayesian inference. Many of these methods are designed for the discovery of long motifs commonly found in prokaryotes, rather than the shorter motifs more common to eukaryotes. These methods might also fail to uncover globally optimal solutions since some form of local search is used in these methods [5]. In contrast, word-based methods describe motifs as word strings or oligonucleotides with degenerative bases. These methods employ exhaustive enumeration to count the frequency of oligonucleotides and identify over-represented oligonucleotide sequences from input promoters. This method is more suitable for identification of the shorter length motifs found in eukaryotic genomes, and is also guaranteed to find globally optimal solutions [5]. A word-based algorithm, Oligo-Analysis, developed by van Helden et al, was proved useful in discovering yeast motifs, but was limited to relatively simple motifs without much degeneration [6]. By contrast, the algorithm Yeast Motif Finder (YMF) allows searching for degenerative motifs containing the wobble bases, r(A|G), y(C|T), s(C|G), and w(A|T), or motifs with spacer n in the middle, but the maximum number of wobble bases in a motif is typically restricted to 2 [7]. Two other algorithms, Discriminating Word Enumerator (DWE) and Discovery of Rank Imbalanced Motifs (DRIM), also search for words with restricted number of degenerated bases [8], [9]. Yet another algorithm, Weeder, searches for motifs ranging from 6–12 bp, with 1–4 mismatches [10]. However, to our knowledge there is yet to be a program that enumerates motifs with all possible wobble bases without limiting their numbers in a single motif.

As more complete genome sequences are available, comparative genomics can be used for motif discovery. These methods aim to identify evolutionarily conserved non-coding sequences via sequence alignment, usually within the promoter region of homologous gene sets from related species. Such a strategy has been used to discover motifs from yeast, *Drosophila*, and mammals [4], [11], [12]. These studies show that many conserved motifs tend to have their positions biased towards the transcriptional start site (TSS), supporting the view that they are *bona fide* motifs.

Here, we describe an algorithm termed MotifIndexer that is based on word counting strategy. We have successfully used this algorithm to identify motifs from promoters of all predicted *A. thaliana* genes and of selected stress-induced co-expressed genes [13]. We demonstrate a new comparative genomics method to discover potential DNA regulatory elements by identifying motifs with conserved positional bias in both the Arabidopsis and the rice genomes. Significantly, the method does not depend on alignments of homologous gene promoter sequences.

## Results

### Indexing the Presence of 8-mer Oligos in the *A. thaliana* Genome and Stress Induced Co-expressed Gene Cluster

In word-counting based motif discovery algorithms, the format used to represent motifs is usually limited by search space volume. To balance between motif length and motif complexity, we chose two different motif formats. The first considers 8-mer oligos, with all possible combination of A, C, G, T, r(A|G), y(C|T), s(C|G), w(A|T), m(A|C), k(G|T), or n(A|C|G|T). The 214,358,881 or 11^8^ oligos in this format cover almost all versatility in terms of nucleotide degeneration. Only wobble bases representing three different nucleotides will be missed in this format. The second format extracts 12-mer oligos, with all possible combination of A, C, G, T, or n. This format, with limited complexity in degeneration, allows characterizing motifs up to 12 bp, and the total number of oligos is 244,140,625 or 5^12^.

**Figure 1:**
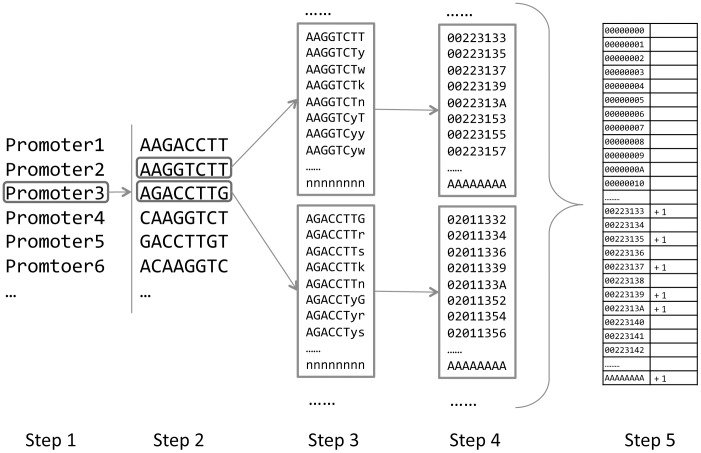
Five steps to count the number of promoters harboring each 8-mer oligos within a selected group of promoters.

For every 8- or 12-mer oligo, we developed an algorithm to efficiently catalog the number of promoters harboring it within the *A. thaliana* genome, or within any selected group of promoters. The procedure for cataloging the 8-mer oligos is shown in Figure 1. The procedure loops through all promoters with iterations. In every iteration, a specific promoter is selected (step 1), all 8-mer oligos without degeneration are extracted (step 2), and listed together with all their degenerative forms (step 3). Next, the collection of 8-mers are translated into numbers according to an undercimal (base-11) positional notation system, in which the nucleotides A, C, G, T, r, y, s, w, m, k, n are converted to digit number 0, 1, 2, 3, 4, 5, 6, 7, 8, 9, and A, respectively (step 4). Then, an array recording the number of promoters harboring these oligos is updated, allowing for an increase of 1 for every appearance (step 5). For 12-mer oligos, the procedure is similar except that a quinary (base-5) positional notation system is used.

Once oligo numbers are cataloged for the whole genome and for promoters of a specific group of co-expressed genes, a pValue is calculated for every 8-mer or 12-mer oligo based on the hypergeometric distribution. This pValue provides a measure for over or under-representation of the corresponding oligo in the selected groups of promoters. Our method guarantees the finding of all 8-mer oligos with pValue smaller than a selected cut-off value. In the following analysis, we focused on oligos existing in fewer than 50% of all the promoters in the genome, which include 75,029,949 8-mer oligos and 242,575,400 12-mer oligos.

To test our algorithm, we selected co-expressed gene groups consisting of 7,424 genes from a previous study that mainly includes Arabidopsis genes that are differentially regulated during biotic and abiotic stress responses [13]. In this analyses, the AtGenExpress stress data set [14] were analyzed using fuzzy k-means clustering, and genes were divided into 178 clusters according to expression patterns, with 22 major clusters (N0-N21; see Figure 2 in [13]). Our analysis here focused on genes induced by both abiotic and biotic stresses (cluster_N0), common stress responsive genes (cluster_N12), and genes induced by pathogen-associated molecular patterns (PAMPs) (cluster_N19).

**Figure 2:**
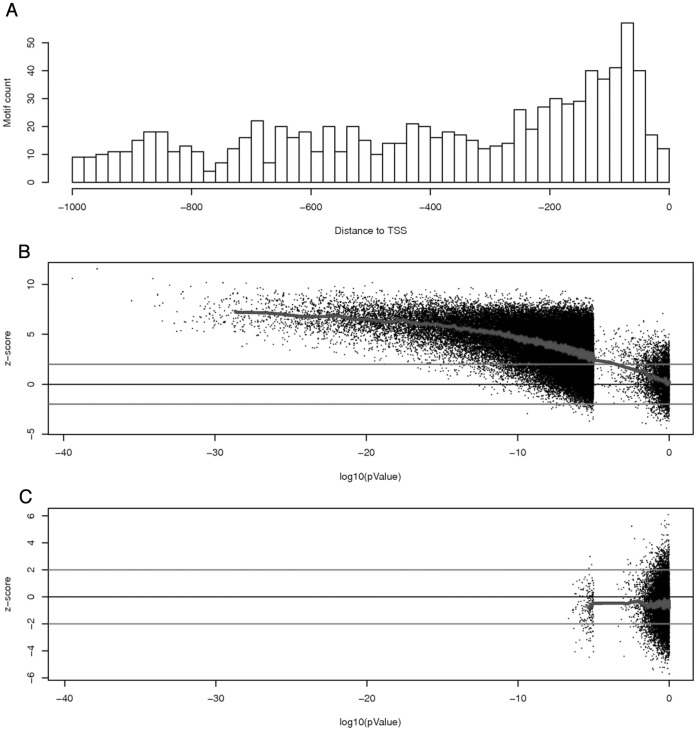
Position bias of motifs. (A) The distribution of the motif “rGTCAAmn" along the promoters in cluster_N0. Distance is relative to the TSS site. (B) A plot for oligos’ pValue against its z-score, from cluster_N0. Each dots represents a oligo. Shown are all oligos with pValue <1E-5, and a 0.03% sampling of those with pValue >1E-5. The blue line shows the trend. (C) A similar plot to B, but with oligos from randomly collected promoters. Note that the trend line is flat around 0.

All 93,138 over-represented 8-mer oligos with pValues < = 1E-05 for cluster_N0 are shown in Table 1, sorted by pValue. The highest scoring two motifs identified were “wrGTCAAm" and “rGTCAAmn", with pValue 7.9E-32 and 1.19E-30 respectively. We noticed that many oligos below these pValues were simply variants of these two primary motifs. To increase the discovery of distinct rather than similar motifs, we used a position-based filter to eliminate variants of major motifs. Once a major motif was selected, its positions along the promoters were marked, and other oligos that share at least 10% of its position were removed. Among the remaining oligos, another major motif was picked from the top candidates with lowest pValue, and its position was marked, in addition to the positions of the previous major motifs. This process was repeated until less than 10 oligos remained. Major motifs identified using this process for cluster_N0 are listed in Table 2.

**Table 1:** Over-represented 8-mer motifs identified based on the pValue in the coexpressed genes promoters of cluster_N0 that are induced by abiotic and biotic stress.

Ranking	Motif	InCluster	In Genome	pValue	q-Value
0	wrGTCAAm	437	12418	3.68E-40	6.91E-33
1	rGTCAAmn	515	16289	1.57E-38	1.47E-31
2	kTTGACyn	515	16297	1.67E-38	1.04E-31
1066	wTGACkTk	361	11232	1.51E-21	2.65E-17
1067	GAAAwkTm	470	16228	1.51E-21	2.66E-17
1068	TwGACnTk	469	16183	1.52E-21	2.66E-17
1300	rrrGTCAr	385	12401	8.84E-21	1.27E-16
1301	ACGCGkww	107	1874	8.93E-21	1.29E-16
1302	rrGTCArw	421	14019	8.95E-21	1.29E-16
1303	AwmGTCAr	315	9380	8.97E-21	1.29E-16
93137	ATwGACTw	186	6508	1.00E-05	2.01E-03
93138	AyGCsTyw	186	6508	1.00E-05	2.01E-03

**Table 2:** Major 8-mer motifs identified based on the position bias filter in the coexpressed genes promoters of cluster_N0 that are induced by abiotic and biotic stress.

Ranking	Motif	In Cluster	In Genome	pValue	Mean position	TSS factor
1	rGTCAAmn	515	16289	1.57E-38	612	11.57
8	AAAGTCww	365	9914	2.15E-34	604	7.89
1067	GAAAwkTm	470	16228	1.51E-21	567	6.05
1301	ACGCGkww	107	1874	8.93E-21	647	5.44
9900	AwAAAAGk	429	15841	2.29E-12	537	3.08
9048	GAATwwTr	437	16150	1.03E-12	527	2.2
6996	wCACGynk	354	12129	9.07E-14	569	5
11205	AATTArTw	404	14760	6.76E-12	521	1.54
13307	ATAAwATA	392	14328	2.84E-11	521	1.53
16102	kACGACyn	174	5127	1.37E-10	570	3.25
23351	AACAAAAA	392	14735	2.28E-09	527	1.99
51838	sACGCrCk	57	1307	3.73E-07	641	3.67
21072	AwTCAAAG	206	6546	1.05E-09	552	2.65
58106	sAAGACTw	153	4899	7.10E-07	603	4.72
84420	TGrCCGCs	26	449	4.88E-06	635	2.35

The over-represented oligos can also be sorted by their fold enrichment in the group of selected promoters relative to the whole genome. For example, in cluster_N0, among the oligos with similarities to the major motifs, many showed higher fold change enrichment than the major motifs themselves. Table 3 lists such oligos, grouped by the related major motifs. However, fold change enrichment is a measure prone to false discovery according to our permutation experiment (data not shown), therefore pValue will be used in the following analysis.

**Table 3:** Major 8-mer motifs ranked by fold-change enrichment in the coexpressed genes promoters of cluster_N0 that are induced by abiotic and biotic stress.

Related Major Motifs	Motifs	Cluster Size	In Cluster	In Genome	Fold change Enrichment	pValue	Mean Position	Z-score for TSS
rGTCAAmn, AAAGTCww	CTTTGACC	712	64	1161	2.6	4.81E-12	676	4.95
	AAAGTCAA	712	203	4185	2.3	7.33E-31	629	6.65
	AGTTGACy	712	100	2096	2.2	4.28E-14	598	3.47
GAAAwkTm, GAATwwTr	GAAAAGTC	712	100	1704	2.8	2.93E-20	679	6.45
	GAAAAGTm	712	186	4292	2.0	3.83E-22	630	6.49
	GAAAwTTC	712	136	4231	1.5	8.08E-07	602	4.18
ACGCGkww, ACrCGnkk	ACGCGTTA	712	22	238	4.4	9.87E-09	549	0.75
	AACCGCGT	712	24	311	3.6	7.00E-08	763	4.45
	AACGCGTy	712	38	493	3.6	1.13E-11	633	2.84
	AArCGCGT	712	54	841	3.0	7.81E-13	706	5.36

### Position Bias of Top Motifs

Typically, promoter motifs show position bias towards the transcription start sites (TSS), a feature that has been used as supporting evidence for *bona fide* motifs in many studies [7], [15], [16], [17]. In our study, top motifs from most of the clusters also have their positions bias towards the TSS. For example, Figure 2A shows the distribution of the motif “rGTCAAmn" along the promoters in cluster_N0. The motif density is much higher in the region 0 to 200 base pair (bp) upstream of the TSS site than in other region.

While the distribution plot depicted in Figure 2A is intuitive in showing the bias of individual motif, it is difficult to apply such plots to a large number of oligos at the same time. Therefore, we used a z-score based on the uniform distribution to mathematically describe such bias. We assume oligos without selection pressure (non-motifs) distribute evenly along the promoter without preference. Since all promoters used in this analysis are 1000 bp in length, the mean position for evenly distributed 8-bp oligos should be at position 497. For an oligo with n instances within the promoters, the variance of their means should be (993^2−^1)/n. Thus, for any 8-mer oligo with n instances within any selected promoters, a z-score is calculated as:
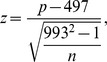



where p is the average position location. For orientation, we describe p = 1 as the position at −1000 relative to TSS, while p = 993 is the position immediately adjacent to TSS.

Evenly distributed oligos should have a z-score close to 0. A large positive z-score indicates the oligo has a higher occurrence in the region between 0 to −500 relative to the TSS than in the −500 to −1000 region. That is, it has its positions biased towards the TSS. The mean position and z-score for the top motif from most clusters are listed in Table S1. It is apparent that many of them have a large z-score, indicating a position bias of top motifs towards the TSS.

### Identification of Major 8-mer Motifs from Different Clusters of Coregulated Stress Responsive Genes

In our analysis, we also observed a correlation between small pValues and positive z-scores for co-expressed stress responsive genes. Such correlations for genes in cluster_N0 are illustrated in Figure 2B. To determine the significance of this observed correlation among co-expressed genes, we analyzed randomly selected promoters with the same size cluster as N0. Not only was the number of oligos with small pValues much smaller, but the z-score for the oliogs also averaged around 0 without much bias (Figure 2C). This result met our expectations, since no motif should be enriched in these randomly selected promoters.

To evaluate the false discovery rate (FDR) in our algorithm, we repeated the analysis on 100 groups of randomly selected promoters, with 50 promoters in each group. The number of oligos resulting from these analyses should be similar to the number of false positive oligos identified in an actual experiment. On average, each group had 1.76, 17.8, or 180 oligos (before position-based filter) with pValue smaller or equal to 1E-7, 1E-6, and 1E-5, respectively. These numbers are close to the expected value: 75,029,949 X pValue/4. Among the 180 oligos with pValue < = 1E-5, only 0.49 oligo has its TSS z-score larger than 3. Analysis on groups of promoters with size 200 or 500 returns slightly (∼10%) more oligos that we would consider to be false positive. Based on these results, we choose the cutoff values in our analysis to minimize the number of false discovered oligos while maximizing the number of *bona fide* motifs.

We used two criteria for qualifying an oligo to be a potential motif. The first one is based on the pValue cut off, and the second one is based on the z-score cut off. For 8-mer oligos, we took all those with pValue <1E-7, and those with pValue between 1E-5 and 1E-7 and z-score >3 as potential motifs. It should be noted that, though the pValue and z-score show correlation, these are two different measurements and are independent of each other. Therefore, motifs qualifying for both criteria should have high confidence to be a true motif. Using these criteria, we identified all 8-mer motifs that are over-represented in various co-expressed gene clusters (Table 4 and Table S2). Among these, cluster_N11 had 2,089 oligos (before position-filter) with pValue <1E-7, while all others had more than 9,000 oligos fulfilling such criteria. Since the number of expected false positive oligos was around 2, the false discover rate would be less than 0.1%. In addition to the identification of known motifs, our analysis revealed 32 putative new motifs.

**Table 4:** Major 8-mer motifs identified for coexpressed genes from various clusters.

Cluster	Cluster Size	Motif	In cluster	In genome	pValue	Mean position	z score for TSS factor	Similar to motif	Motif sequences
**Genes induced by pathogen elicitors and PAMPs**									
**N19**	217	TTGACTTy	101	5626	1.63E-24	624	5.06	WBBOXPCWRKY1	TTTGACy
	217	kGTCAAmn	154	16388	4.85E-11	559	3.16	WBBOXPCWRKY1	TTTGACy
	217	**sCGTTkAn**	93	7797	1.50E-10	598	3.42	n/a	
	217	**TCGAATTk**	51	3420	1.20E-08	542	1.02	n/a	
	217	CACCwmCC	32	1941	1.13E-06	712	4.38	BOXLCOREDCPAL	ACCwwCC
	217	**AGTCkTCG**	21	1032	4.13E-06	701	3.17	n/a	
**Genes induced by various biotic and abiotic stresses**									
**N0**	712	rGTCAAmn	515	16289	1.57E-38	612	11.57	WBBOXPCWRKY1	TTTGACy
	712	AAAGTCww	365	9914	2.15E-34	604	7.89		
	712	GAAAwkTm	470	16228	1.51E-21	567	6.05	GAAATTT	GAAATTT
	712	GAATwwTr	437	16150	1.03E-12	527	2.2		
	712	ACGCGkww	107	1874	8.93E-21	647	5.44	CGCGBOXAT	vCGCGb
	712	ACrCGnkk	310	9212	1.85E-20	602	6.95		
	712	**kACGACyn**	174	5127	1.37E-10	570	3.25	n/a	
	712	**sACGCrCk**	57	1307	3.73E-07	641	3.67	n/a	
	712	**wCACGynk**	354	12129	9.07E-14	569	5	n/a	
	712	**AwAAAAGk**	429	15841	2.29E-12	537	3.08	n/a	
	712	**AATTArTw**	404	14760	6.76E-12	521	1.54	n/a	
	712	**ATAAwATA**	392	14328	2.84E-11	521	1.53	n/a	
	712	AACAAAAA	392	14735	2.28E-09	527	1.99	ANAERO1CONSENSUS	AAACAAA
	712	AwTCAAAG	206	6546	1.05E-09	552	2.65	T-box promoter motif	ACTTTG
	712	**sAAGACTw**	153	4899	7.10E-07	603	4.72	n/a	
	712	**TGrCCGCs**	26	449	4.88E-06	635	2.35	n/a	
**N12**	197	mCGCGTnn	87	3760	8.59E-32	709	7.95	CGCGBOXAT	vCGCGb
	197	GCGCGTsm	15	565	1.46E-06	723	3.08		
	197	**rCGTGTnn**	136	11958	2.02E-21	633	7.01	n/a	
	197	**CCGTGTnk**	32	2089	6.21E-07	671	3.32	n/a	
	197	CCACGyGs	31	1197	4.29E-12	720	4.42	Agris_GBF1/2/3 BS in ADH1	CCACGTGG
	197	rCCGACny	50	4077	2.97E-07	638	3.62	DRECRTCOREAT	rCCGAC
	197	wAATATCk	91	9177	1.93E-08	544	1.56	EVENINGAT	AAAATATCT
	197	**AAAArAGA**	133	15989	2.44E-08	572	3.41	n/a	
	197	AwwTTGAC	87	8781	5.25E-08	536	1.19	WBOXATNPR1	TTGAC
**Genes downregulated during stress**									
**N18**	465	nGGCCCAn	304	8287	2.14E-77	854	26.74	UP1ATMSD	GGCCCAwww
	465	mAGCCCAn	223	6964	2.67E-39	806	18.83	SITEIIATCYTC	TGGGCy
	465	AAACCCTr	181	5612	1.55E-30	786	14.54	UP2ATMSD	AAACCCTA
	465	**CCGGnnTn**	222	11462	1.50E-09	571	4.28	n/a	
**N14**	302	AGGGTTTw	152	5787	8.26E-40	870	18.41	UP2ATMSD	AAACCCTA
	302	**ArGCCCrT**	104	4709	2.78E-19	828	13.2	n/a	
	302	**nCCGGAnn**	175	12653	1.02E-12	604	5.81	n/a	
	302	**rGCCCArw**	115	7141	2.54E-11	763	11.64	n/a	
	302	**GGTTsGGw**	59	2773	5.83E-10	676	4.8	n/a	
	302	nCGrCGkn	149	10534	8.55E-11	588	4.49	CGACGOSAMY3	CGACG
	302	**AGGsCTTm**	35	1431	9.86E-08	652	3.17	n/a	
	302	**rGGTTTmw**	165	11494	4.66E-13	681	9.33	n/a	
	302	**sGwTTTAs**	122	8621	2.11E-08	627	5.36	n/a	
**N2**	1292	**AwTGGsCy**	419	7451	7.03E-18	709	17.61	n/a	
	1292	nGsCCCAn	522	10089	1.11E-15	685	17.76	UP1ATMSD	GGCCCAwww
	1292	**CCkGTTTr**	206	3603	6.65E-09	654	8.13	n/a	
	1292	**rnACGACr**	395	7940	8.71E-09	581	6.09	n/a	
	1292	GATAAGnn	627	13746	2.89E-08	595	9.33	IBOX	GATAAG
	1292	CTCACTsw	193	3405	4.43E-08	544	2.04	SORLIP5AT	GAGTGAG
	1292	**rkGGACCn**	262	5081	4.73E-07	555	3.16	n/a	
**N5**	820	AwTGGGCy	259	6146	2.16E-20	738	15.86	SITEIIATCYTC	TGGGCy
	820	rrCCGTTr	196	4190	1.09E-19	669	9.43	MYBCOREATCYCB1	AACGG
	820	GCGsGArm	86	1689	1.42E-10	656	5.21	E2F1OSPCNA	GCGGGAAA
	820	AGwGwGwG	344	10773	2.48E-09	609	8.52	CTRMCAMV35S	TCTCTCTCT
	820	**AmCCGAAC**	79	1843	1.75E-06	639	4.82	n/a	
	820	rGGsTTTw	345	11207	1.78E-07	637	10.24	UP2ATMSD	AAACCCTA

(Putative new motifs are marked bold).

Genes in cluster_N19 are induced by pathogen elicitors/effectors or PAMPs and have roles in defense. As expected, two W-Box like motifs “TTGACTTy" and “kGTCAAm" are highly over-represented in this cluster of co-expressed genes (Table 4). WRKY transcription factors have been shown to bind to the W-Box motif during defense response [18], [19]. Another motif in cluster_N19 with moderate pValue, which nevertheless showed strong position bias is “CACCwmCC" (Table 4). This motif is similar to the BOXLCOREDCPAL motif “ACCwwCC", which is the binding site for DcMYB1, a carrot MYB transcriptional activator of the DcPAL2 gene in response to elicitor treatments [20]. The BOXLCOREDCPAL motif itself is not over-represented. The genes in cluster_N19 with the “CACCwmCC" in their promoters are predicted to function in secondary metabolism processes, especially phenylpropanoid synthesis. This indicates a MYB regulated signaling pathway is activated upon PAMP treatment to synthesize metabolites that facilitate defense responses. Interestingly, we also identified a motif “sCGTTkAn" with position bias (Table 4) that has no similarity to any known motifs in the plant motifs databases Agris and PLACE [21], [22]. We hypothesize that a yet to be identified transcription factor class will bind to the “sCGTTkAn" motif and that this motif will most likely be involved in the regulation of elicitor/effector or PAMP induced gene expression.

Genes in cluster_N0 are induced by biotic and abiotic stresses. The over-represented motifs “rGTCAAm" and “AAAGTCww" (Table 4) are related to the W-Box motif. By their presence they might explain the induction of W-Box containing genes by PAMPs. The motif “ACGCGkww" and “ACrCGnkk" are related to the CGCG motif with the core sequences as “ACGCGT". The Ca^2+^/calmodulin-binding transcription factor AtSR1 (also known as CAMTA3) has been shown to bind to the CGCG motif within the promoter of the EDS1 gene acting as a repressor of its expression [23]. EDS1, an important pathogen responsive gene [24], is involved in regulating the levels of the defense molecule salicylic acid (SA). EDS1 is included in the cluster_N0 gene set and the identification of “ACGCGkww" and “ACrCGnkk" motifs within the promoters of other cluster_N0 genes indicates that they may also be regulated by AtSR1 or related transcription factors, and that their functions might be EDS1-independent. Our analysis of genes in cluster_N0 also identified a motif “GAAAwkTm" (Table 4) that is related to the “GAAATTT" motif. “GAAATTT" is the binding motif for CBP60g and SARD1, two transcription factors with partially redundant role in SA signaling [25], [26]. Many of the CBP60g and SARD1 downstream genes are enriched in cluster_N0. In addition, AT rich motifs such as “AwAAAAGk", “AATTArTw", “AATAwATA", and “AACAAAAA" are also over-represented in genes of cluster_N0 together with potentially new motifs “kACGACyn", “sACGCrCk", “AwTCAAAG", and “TGrCCGCs" (Table 4).

Genes in cluster_N12 are induced by a variety of abiotic or biotic stresses. These genes are often viewed as common stress responsive genes. In this cluster, motif “mCGCGT" and the related motifs “ACrCGy" and “rCrCGkmm" are highly represented (Table 4), all three of which are similar to the CGCG motif and the rapid stress responsive element “CGCGTT" [27]. They are also similar to the CM2 motif “CCGCGT" within the ZAT12 promoter, which is the binding site for transcription factor CAMTA3 [28]. ZAT12 is included in the cluster_N12 gene set. This indicates that the CGCG motif not only plays a role in SA-responsive gene expression but also in mediating general stress responsive gene expression. Other over-represented motifs in cluster N12 are “AAAArAGA", “GwCCGACk", “CCACkwGG", and “TAAGGCGk" (Table 4). Similarly, genes in cluster N11 are also induced by various stress, but to a lesser intensity, and the over-represented motifs in these genes include “mCGCGTnn", “rGTCAAAs", “GACTTTkn", and “CGTGTkwn" (Table S2).

Promoters of genes from several other clusters used in this study are induced by the plant stress hormone abscisic acid (ABA) at early time points (N3) or late time points (N9, N10, N13), and also by other abiotic stresses. As reported earlier [13], the G-Box and related motifs were over-represented in these clusters: “GmCACGTr" in N3, “GmCACGTn" in N9 and N13, and “kmCACGTn" in N10 (Table S2). The G-Box motif “GmCACGTs" is also highly represented in cluster N1 genes that are induced by light. Interestingly, this G-Box motif in cluster_N1 shows the highest position bias among all G-Box motifs in various clusters (Table S2).

Our analysis also identified many motifs from N18, N14, N2, and N5 cluster genes that are down-regulated during stress (Table 4). Cluster_N18 includes many genes encoding ribosomal proteins. Three motifs “nGGCCCAn", “mAGCCCAn", and “AAACCCTr" are over-represented in this cluster with highly-biased positioning (Table 4). Two of them show similarities to known motifs UP1ATMSD “GGCCCAwww" and UP2ATMSD “AAACCCTA" [29]. Cluster_N14 is enriched with genes encoding RNA helicases and these genes are slightly up-regulated by cold stress. Multiple motifs are identified in this cluster including “AGGGTTTw", “ArGCCCrT", “nCCGGAnn", “rGCCCArw", “GGTTsGGw", and “nCGrCGkn" (Table 4). Cluster_N5 contains many cell cycle related genes and its over-represented motifs includes “AwTGGGCy", “rrCCGTTr", “GCGsGArm", “AGwGwGwG", and “AmCCGAAC" (Table 4). Genes in cluster_N2 are highly down-regulated by pathogen stress and includes over represented motifs “AwTGGsCy", “nGsCCCAn", “CCkGTTTr", and the I-box motif “GATAAGnn" (Table 4). Interestingly, the above described clusters share the common motif, “GGCCCA". In their majority, the motifs described above are unique and have not been previously identified. These results indicate that yet to be identified transcription factors and signaling pathways may be responsible for regulating expression of genes in these clusters.

### Identification of Over Represented 12-mer Motifs in Stress-responsive Gene Clusters

It has been reported that transcription factor binding motifs from eukaryotes are 6–10 bp long. Our 12-mer motif analysis supports this notion. By including letter “n" in the motifs, our algorithm revealed that the length of most core motifs is less than 9 bp (Table S3). For example, major 12-mer motifs identified from cluster_N18 are “nnTGGGCCnnnn", “nnAAGCCCAnnn", and “nnAAACCCTAnn" (Table S3), whose sequences are almost identical to the three major 8-mer motifs “nGGCCCAn", “mAGCCCAn", and “AAACCCTr" (Table 4). The trailing letter “n" at both ends of these motifs indicates the core sequences are 7 or 8 base pairs long. Similarly, major 12-mer motifs in cluster_N0 are “TTGACTTnnnnn", “GTCAACnnnnnn", “GTCAAAnAnnnn", and “AnCGCGTnnnnn" (Table S3).

One exception is found among motifs identified from cluster_N7. Genes in this cluster are down-regulated by stresses and mainly expressed in xylem tissue of roots. The motif “CGTGnGnGGCAC" is over-represented in this cluster, with highly biased positioning towards TSS.

Collectively from this analysis, we conclude that the analysis on 8-mer oligos captures most motifs, while the analysis on 12-mer complements and enforces the information gained from 8-mer oligo analysis and may recover a few additional long motifs.

### Using MotifIndexer to Identify Over-represented Motifs from a Single Microarray Experimental Dataset

We tested if our algorithm could be used to identify over-represented motifs from dataset that are generated from single microarray experiments. The data were extracted from AtGenExpress and include Arabidopsis responses to the bacterial pathogen *Pseudomonas syringae* pv. tomato (*Pst*) expressing avrRpm1 effector. This analysis identified 461 genes that were up-regulated more than two-fold in *Pst* expressing *avrRPM1* after 6 hours compared to the mock control (Dataset ME00331: http://www.arabidopsis.org/portals/expression/microarray/ATGenExpress.jsp). Our analysis of the promoters of these genes using MotifIndexer algorithm are listed in Table S4. This includes “rCGTGTnn", “ATATTwTA", “TCTAGAmr", “wrTTGACn", and “yATTCAAm" motifs. Not surprisingly, these motifs are similar to those found in cluster N0, N12, and N19 (Table 4), which includes majority of abiotic/biotic stresses responsive genes. Thus, these motifs can be expected to play important roles in plant responses to pathogens. The identification of these particular motifs unequivocally demonstrates our algorithm’s competence to realistically identify motifs in single datasets from microarray and/or RNA-Seq experiments.

### Using MotifIndexer to Compare Promoters in Two Different Species of Arabidopsis

*A. lyrata* is a close relative of *A. thaliana*. The coding regions between the two genomes share ∼92% identity, while the promoter regions have ∼85% identity [30]. We hypothesized that these two species share similar co-expressed gene modules, and the corresponding motifs are conserved. We tested if MotifIndexer can be used to identify such conserved motifs by comparing these two genomes. For any given set of promoters from *A. thaliana*, we chose those have orthologous promoters in *A. lyrata* and subjected them to MotifIndexer analysis. The same procedure was carried out for the orthologous promoters in *A. lyrata*. For each 8-mer oligo, pValues from each species were obtained and the larger value of the two was assigned as a conserved pValue to that 8-mer oligo.

The analysis was performed on groups of randomly chosen promoters, or on groups of promoters from co-expressed genes. For 100 groups of 30 randomly chosen promoters, there were on average 1,730 oligos with pValue < = 1E-04 in *A. thaliana*, while only 12 of them were conserved among the two species. Thus, 99% of random noises or false positive oligos can be removed by comparing the two genomes. And less than 1 oligo (0.45 in average) was left if the conserved pValue was set at pValue < = 1E-05. Similar results were obtained for 100 groups of 100 randomly chosen promoters. On the other hand, for promoters from cluster_N0, there were 164,215 oligos with pValue < = 1E-04 in *A. thaliana*, and 75,371 of them also have associated pValue < = 1E-04 in *A. lyrata*. Thus, 46% of the oligos are conserved among the two species. Similar results were also obtained for promoters in other clusters (data not shown). The conserved motifs in cluster_N0 identified via this procedure are listed in Table 5. Since much less false positive oligos were left, we set a cut off conserved pValue at 1E-05. The top 3 motifs, “rGTCAAmn", “GAAAwkTC", and “rmCGCGTw" are similar to the top motifs identified in analysis for *A. thaliana* alone. The other four motifs demonstrate the power of such comparative genome analysis. They share similar pValues and position bias distribution in both species, with conserved pValue between 1E-05 and 1E-08. Comparing with single species analysis, their pValues did not change, but the corresponding cut-off pValue was 2 orders larger. Thus, these motifs that would have been considered less significant in a single species analysis were more significant in a comparative genome analysis. This indicates comparative genome analysis using MotifIndexer can identify novel motifs.

**Table 5:** Motifs identified in cluster_N0 via comparison between *A. thaliana* and *A. lyrata.*

	*A. thaliana*			*A. lyrata*			
Motif	pValue	Mean Position	z score forTSS factor	pValue	Mean Position	z score forTSS factor	Conserved pValue
rGTCAAmn	1.64E-38	554	5.23	2.91E-35	557	5.38	2.91E-35
GAAAwkTC	5.34E-22	605	6.39	1.29E-17	595	5.6	1.29E-17
rmCGCGTw	7.78E-23	613	4.07	2.60E-15	628	4.08	2.60E-15
wCnACGAm	1.67E-08	561	3.9	2.02E-09	573	4.74	1.67E-08
TTGAATwk	1.72E-08	567	4.76	2.64E-08	547	3.3	2.64E-08
ACrCGCTn	1.31E-07	538	1.07	2.33E-07	580	2.36	2.33E-07
CGkACGmC	6.57E-06	485	−0.29	2.61E-06	472	−0.52	6.57E-06

### Comparison of MotifIndexer with Weeder and Amadeus Word-based Motif Finders

We compared results obtained from MotifIndexer with two word-based motif finding software Weeder and Amadeus [10], [31]. Weeder had been shown to out-perform other motif discovery tools [3]. Using Weeder version 1.4.2, the top 15 potential motifs were identified from clusters_N0, _N19, and _N18. Similarly, motifs were also identified from these clusters using Amadeus V1.2 with default settings. The results were compared to those (8-mer motifs) from our MotifIndexer algorithm (Table 6). All three programs identified the same highest ranking motifs, e.g., the W-Box related motifs in cluster_N0 and _N19, and the “nGGCCCAn" motif from cluster_N18. Several lower ranking motifs were identified by MotifIndexer and Amadeus, but not by Weeder, such as the CGCG related motif “ACGCGkww" motif from cluster_N0, and the “TCGAATTk" motif from cluster_N19. There were several motifs only identified by MotifIndexer, such as the “CACCwmCC" motif from cluster_N19, and the “GAAAwkTm" motif from cluster_N0 that are known to play a role in SA related defense response [25], [26]. On the other hand, Amadeus also recovered several distinct motifs from cluster_N0 and N18, most of which did not have position bias in the promoters. At the same time, Weeder identified several CG rich motifs from cluster_N0 and _N18 that were not identified in MotifIndexer, but they only exist in a small number of promoters in both clusters (data not shown). These results indicate that, for MotifIndexer and Amadeus, while both identify more motifs than Weeder, they also recovered distinct motifs by themselves.

**Table 6:** Comparison of 8-mer motifs identified from MotifIndexer vs Weeder and Amadeus.

Cluster	Motif identified by MotifIndexer	Motifs identified by Weeder	Motifs identified by Amadeus (**)
N0	rGTCAAmn, AAAGTCww	TTGACT, TTGACTTT, GTTGAC, GACTTT, GACTTTTC, TTGACC, TGACTT, CGTTGACT, TGACTA	CwwrGTCAAm
	GAAAwkTm, GAATwwTr (*)		
	ACGCGkww, ACrCGnkk		ACGCGkTTw
	kACGACyn, sACGCrCk, wCACGynk (*)		
	AwAAAAGk, AATTArTw, ATAAwATA, AACAAAAA, AwTCAAAG (*)		
	sAAGACTw (*)		
			kACTTTTTmA, mrvACkTTTA, TATTdCAATw, AmTwAwTTGC (*)
		GGCGTACGCG, CGCGGCCAGG, ACGCGCGT, GGGCCGCC, AGGGCGGCCT, GGACGCCC, GCTGCCCCCG, GGCTGCCGCG (*)	
N19	TTGACTTy, kGTCAAmn	GTTGAC, GTTGACTT, CGTTGACTTT, TTGACT, CGTTGACT, GTTGACTTTT, TGACTT, TTGACTTT, GTTGACTTTG, GACTTT, GCGTTGAC, GCGTTGACTT, TTGACC, AGTTGACT, AGCGTTGACT, ATTGAC, GGTTGACT, AGTTGACTTT	AdrGTCAAAb
	sCGTTkAn (*)		
	TCGAATTk		whTCGAAkTT
	CACCwmCC (*)		
	AGTCkTCG (*)		
			krAnAATTsA (*)
N18	nGGCCCAn	TGGGCC, AGGCCC, CAGGCCCA, CTGGGCCT, AGGCCCAT, CGGCCCAG,	mrGCCCA
	mAGCCCAn	GCCCAT, TTGGGC, AAGCCC	mrGCCCA
	AAACCCTr		AAACCCTAr
	CCGGnnTn (*)		
		GCGCCAGGGC, CCGCAGGGGC, AAGGCCCG, GCGCTTGCGC, CAGGCGCTGC, AGCCCGGGGC (*)	
			ArCrrkAGTw, mArCGrCATC (*)

(*)denotes motif identified by a single program.

(**)Amadeus represents motifs as position weight matrix. For easy comparison, they are transformed into oligo formats.

### Genome Wide Discovery of Position Biased Motifs in the Arabidopsis and the Rice Genomes

The search for motifs with position bias towards TSS can also be applied to all promoters in a genome. To this end, we applied our algorithm to both the Arabidopsis and the Rice genomes. For Arabidopsis, we included the promoters from 17,461 genes that have a 5′ untranslated regions (UTR) of at least 50 bp in length. The promoters chosen extended 1000 bp upstream of the 5′ UTR. For rice, we included 22,493 genes’ promoters with the same criteria.

For both Arabidopsis and rice, we calculated the z-score for 8-mer motifs with less than or equal to 2 base pairs as degenerative, with the degenerative bases limited to r, y, s, w, or n. This analysis resulted in 28 oligos with obvious position bias in both the Arabidopsis and the rice genome (Table 7). Of these 28 oligos, 19 bear similarities to or share binding sites with known motifs. Among these known motifs, some functions in house-keeping gene expression such as TATABOX1 and UP1ATMSD, while others function in stress responses such as ABFs and TGA1 binding sites. The other 9 oligos do not have similarity to known motifs from the databases of PLACE and Agris. However, because their position biases are conserved among both the Arabidopsis and the Rice genome, we hypothesize that they are novel potential motifs that are regulated during yet to be identified biological pathways.

**Table 7:** Motifs with shared position bias between Rice and Arabidopsis.

			Arabidopsis			Rice		
Motif	Simlar to	Similar to Motif	Instances	Mean position	z-score	Instances	Mean position	z-score
GGCCCAnn	Place_UP1ATMSD	GGCCCAwww	7625	689	58.58	19856	667	83.652
AGCCCAnn	Place_SITEIIATCYTC	TGGGCy	7646	633	41.393	12307	604	41.213
CACGyGnC	Agris_ABFs binding site	CACGTGGC	2790	651	28.288	5551	609	29.027
TCTCTCTy	Place_CTRMCAMV35S	TCTCTCTCT	7243	585	26.014	10033	587	31.265
GGsTTTTn	Agris_TELO-box promoter	AAACCCTAA	8292	566	21.993	7733	574	23.745
**AAACCGnn**	n/a		10210	561	22.379	11960	550	20.223
TGACGyGn	Agris_TGA1 binding site	TGACGTGG	1954	612	17.645	5024	570	18.142
**CCGrnCCG**	n/a		1386	624	16.445	4294	599	23.201
AAAAsGCs	Place_CDA1ATCAB2	CAAAACGC	1828	602	15.667	2317	594	16.209
CTATAAAw	Place_TATABOX1	CTATAAATAC	4636	560	15.055	5856	555	15.592
TAAAsCCn	Place_UP2ATMSD	AAACCCTA	5531	555	14.933	4131	568	15.952
AnnCGACG	Place_CGACGOSAMY3	CGACG	3697	563	14.086	7802	558	18.743
GCGCGnGn	Place_CGCGBOXAT	vCGCGb	1033	621	13.937	11696	574	29.001
**sCCGTTTn**	n/a		2183	581	13.655	3966	561	14.155
GnCACGTw	Agris_ACE promoter	GACACGTAGA	2069	582	13.415	4007	551	11.984
GnCCGTTr	Place_MSACRCYM	AGACCGTTG	1466	585	11.785	2546	576	13.926
TAAATAss	Place_TATABOX1	CTATAAATAC	2827	560	11.645	3238	555	11.418
GGwCCCAC	Place_SITEIIBOSPCNA	TGGTCCCAC	431	642	10.471	2413	590	15.999
**GCCTwTAn**	n/a		2550	556	10.313	3480	562	13.361
**CsGTyCGA**	n/a		822	593	9.547	1793	616	17.525
**CGCGTTwA**	n/a		318	606	6.794	360	611	7.508
**GyGGGGTs**	n/a		326	597	6.286	2423	596	17.031
CCGACCsA	Place_DRE2COREZMRAB17	ACCGAC	326	593	6.07	905	594	10.126
**CGGGTCAA**	n/a		283	600	6.061	309	599	6.255
TGACGTCA	Place_PALINDROMICCBOXGM	TGACGTCA	259	596	5.556	270	593	5.514
ACCCrCCC	Place_ACIPVPAL2	CCCACCTACC	228	595	5.161	1323	642	18.419
**TGGGGCCw**	n/a		200	590	4.592	1031	590	10.357
AGCGrGCC	Place_BS1EGCCR	AGCGGG	124	607	4.261	780	604	10.397

(Putative new motifs are marked in bold).

In addition to identifying motifs that show strong position bias in both Arabidopsis and rice, we identified motifs with position bias in only one species, either Arabidopsis (Table S5) or rice (Table S6). In Arabidopsis, 12 out of the 28 biased motifs have shared binding sites with known plant motifs, while in rice 21 out of 70 motifs are known. The remaining motifs are potential novel motifs with possible monocot or dicot distinguishing differences, although the confidence values for these motifs are less pronounced when compared to the candidate motifs shared by both species. However, comparison with other plant species should improve the confidence value.

## Discussion

Here, we describe an algorithm that identifies all over-represented 8-mer oligo in groups of promoters that meet a predetermined pValue cut off, except for those oligos with wobble bases representing three nucleotides. It should be noted that wobble bases representing three nucleotides are rarely used for motif discovery [21], [22]. The motif discovery method described here is based on counting oligos (words) without allowing for mismatches. This is distinct from other methods such as Weeder that includes mismatches [10]. We find our method to be more suitable in part because it has been shown that mutations in one or two critical bases in a motif can abolish its binding affinity to transcription factors [18], [23], [32]. In addition, our method expands on and appears superior to a previously described exact counting method [7] because it greatly expands the oligo coverage in terms of degeneration. Comparison of our MotifIndexer with Weeder and Amadeus, we found our program performs similarly to Amadeus [31], while both recovered distinct motifs.

As a measurement for over-representation of oligos, a pValue was calculated for every oligo based on the hypergeometric distribution [33]. In the 8-mer motif analysis, only 75,029,949 oligos that exist in less than half of the genome’s promoters were considered. Among these oligos, we expected that ∼50% are present in a frequency larger than expected, or over-represented, in any cluster. Only these 50% oligos were used for pValue calculation. From this we further removed reverse complemented counterparts. Thus, the expected number of false positive oligos is 75,029,949×pValue/4. Our analysis on groups of randomly selected promoters fits well with this expectation. Based on this, we chose pValue cut-off of 1E-07 for our analysis, thus limiting the total number of false positive oligos to 2 in average. This cut-off worked well for co-expressed gene clusters with large number of genes. The clusters used in our analysis usually have more than 100 genes and many motifs with pValues smaller than 1E-07 were identified. However, for smaller clusters, i.e. those with 20–50 genes, true motifs might have their pValue larger than 1E-07, and a larger pValue cut-off is needed. In this case, comparative genomics can be used to further remove false positive oligos. *A. lyrata* is a close relative of *A. thaliana*. The homologous promoters from these two species share similarity levels between 75% and 95%. Under these similarity levels, the true motifs are expected to be conserved between the two species, while the false positive oligos are not. Since MotifIndexer calculated a pValue for every possible 8-mer motif, it can be easily adapted for comparative genomic analysis, as shown for cluster_N0 promoters (see Table 5). By comparing two genomes, the pValue cut-off can be lowered to 1E-05, and more conserved motifs will be recovered. We also found that our MotifIndexer program can be used for motif identification based on gene co-expression networks (data not shown). This indicates the versatility of MotifIndexer, a distinct advantage when compared to other motif finding programs.

Our method uses a z-score to describe oligos position bias within the selected promoters as another independent measurement of likelihood of identifying a *bona fide* motif. Previously the position bias for individual motifs has been depicted by a plot of motif density vs. relative position to the TSS [4]. In several studies, position bias was also measured by dividing promoters into several windows of predetermined width and then then searching for over-represented motifs within these bins [15], [34]. Recently, Yokoyama et al. developed a motif-positional function to measure spatial preferences at fine-scale resolution [17]. Our z-score method based on uniform distribution provides an alternative way to describe motif position bias at fine-scale resolution. It can be easily applied on a large number of oligos at the same time. By combining pValues and z-scores, our method provides a simple but powerful scoring system for the oligos. By applying this scoring system to randomly selected groups of promoters, cut-off values for *bona fide* motifs can be easily determined (see Figure 2). However, if a motif has position bias in the middle of the promoters, such as around −500 bp in promoters of 1,000 bp long, our program will not identify such bias.

We applied a position based filtering system to pool major motifs with their related degenerate motifs, which enabled us to differentiate motifs occurring at unique binding sites. By applying this scoring and filtering system, we identified many known/unknown motifs with pValue less than 1E-7, as well as some motifs with moderate pValue but obvious position bias. Some of the previously not reported motifs bear similarities to known motifs, increasing confidence of them being true motifs. We do recognize that the filtering method applied here might mask motifs sharing partial binding sites.

Our analysis with 8-mer oligos guaranteed that those motifs with lowest pValue were found. The subsequent analysis on 12-mer oligos confirmed the length of core motifs to be less than 9 base pair. Besides core motifs, flanking sequences might also be important to determine binding specificity, especially specificity within the same family of transcription factors, i.e. the WRKY transcription factors [18]. Once the core motif sequences are determined, such flanking sequences can be determined by analyzing nucleotide composition for base pairs around the core motif sequences (data not shown). Our analysis can also be modified to study motifs in which interior spaces are inserted, such as “nnnn". However, a trial run did not reveal any meaningful motifs.

We also used position bias to identify potential motifs on genome wide scale using comparative genomics. The examples shown in Table 7 indicate this is a promising tool. Previous applications of comparative genomics for motif finding have been mostly limited to comparing homologous genes of different genomes [12], [35]. Our method is not reliant on homologous gene comparison and thus expands our discovery scope. It is yet to be explored if their close relative species share similar motif bias or not.

In conclusion, we describe a *de novo* motif discovery method which needs no parameter input and are suitable for the identification of over-represented motifs from large-scale transcriptome datasets. We name this method as MotifIndexer. Currently, we are probing our Arabidopsis protein microarrays containing transcription factors [36], [37] to identify factors that bind to novel and known motifs identified in this analysis.

## Materials and Methods

### Clustering Data and Promoter Sequences

The clustering data for co-expressed gene groups were taken from a previous study [13]. In that study, the AtGenExpress global stress data set was analyzed via fuzzy-k means clustering method [14], [38]. Twenty-two major clusters identified from that analysis were extracted and used in this analysis.

Arabidopsis promoter dataset was downloaded from TAIR (ftp://ftp.arabidopsis.org/Sequences/blast_datasets/TAIR9_blastsets/TAIR9_upstream_1000_20090619). The dataset contains the upstream 1000 base pairs for 33,518 genes. The rice upstream sequences were downloaded from the Rice Annotation Project Database (http://rapdb.dna.affrc.go.jp/). The IRGSP/RAP build 5 dataset was used to extract the 1000 base pair sequences upstream of 5′UTR from those genes with 5′UTR longer than 50 bp.

### Indexing Genome and Oligo pValue Calculation

The procedure to catalog the number of promoters containing specific 8-mer or 12-mer oligos is illustrated in Figure 1. We used algorithms written in perl and C++ to carry out the cataloging process. The algorithms have been tested in Linux and Windows system, which can scan ∼18 promoters (1000 bp in length) per minute with an Intel® Core™ i5 CPU M540 @2.53 GHz. 1 G memory is required for the calculation. The MotifIndexer algorithm will be provided through our website (http://dinesh-kumarlab.genomecenter.ucdavis.edu/downloads.html) and upon request for academic use.

For pValue calculation of oligos, we only consider those presented in less than half of genome’s promoters, which includes 75029949 8-mer oligos and 242575400 12-mer oligos. Suppose in a group of selected genes with *M* promoters in total, an oligo presents in *m* promoters among them. And within the *K* promoters in the whole genome, the oligo presents in *k* promoters. If *m* is larger than the expected value, that is *m* > *k* *M/*K*, a pValue is calculated as:
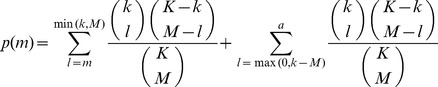
where *a* is the largest integer between 0 and *k* *M/*K* that fulfills the requirement:






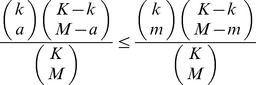


After pValues were calculated for all the over-represented oligos, they were sorted by their pValues. And for any pair of reverse complemented oligos, only one oligo is retained. Then, the selected promoters were scanned for the presence of the oligos to calculate their average position and z-score for TSS. Finally, major motifs were picked as described in the result section.

The process was carried out on selected co-expressed gene clusters, as well as randomly selected promoter groups.

### Promoter Comparison between *A. thaliana* and *A. lyrata*

The genome sequence of *A. lyrata* was downloaded from http://genome.jgi-psf.org/Araly1/Araly1.download.ftp.html. The protein sequences of *A. lyrata* (Filtered Models6) were blasted against the *A. thaliana* protein sequences, and homologous gene pairs were selected based on these criteria: a, having at least 80% identities in amino acid sequences; b, the length of the *A. lyrata* protein is between 0.8 to 1.2 fold of that of the *A. thaliana*; c, potential duplicated genes in *A. lyrata* were filtered out. In total, 19,938 genes from *A. lyrata* were left and their promoters were extracted as 1,000 base pairs up stream of the translation start codon, and the same was done for *A. thaliana*. The analysis was carried out as described in the result section.

## Supporting Information

Table S1 **Top motifs have position bias.**
(DOC)

Table S2 **Major 8-mer motifs identified for coexpressed genes from various clusters.**
(DOC)

Table S3 **Major 12-mer motifs over-represented in stress responsive genes promoters.**
(DOC)

Table S4 **Major motifs identified from Arabidopsis genes induced by** ***Pseudomonas syringae*** **pv. tomato (** ***Pst*** **) (avrRpm1) – a case study on a single microarray experiment.**
(DOC)

Table S5 **Motifs with position bias only in Arabidopsis.**
(DOC)

Table S6 **Motifs with position bias only in Rice.**
(DOC)
