# Supplementary material for: Discovery of Stress Responsive DNA Regulatory Motifs in Arabidopsis
Source: PLoS One. 2012 Aug 13;7(8):e43198. doi: 10.1371/journal.pone.0043198 (PMC3418279; doi:10.1371/journal.pone.0043198)
Supplement: Table S1 — Top motifs have position bias. (DOC) [file pone.0043198.s001.doc]

Table S1. Top motifs have position bias

| **Cluster** | **Cluster**  **Size** | **Motif** | **In cluster** | **In genome** | **pValue** | **Mean position** | **z score**  **for TSS factor** |
| --- | --- | --- | --- | --- | --- | --- | --- |
| N0 | 712 | rGTCAAmn | 515 | 16289 | 1.57E-38 | 612 | 11.57 |
| N1 | 354 | GmCACGTs | 98 | 3199 | 1.15E-22 | 839 | 12.32 |
| N2 | 1292 | AwTGGsCy | 419 | 7451 | 7.03E-18 | 709 | 17.61 |
| N3 | 154 | GmCACGTr | 92 | 3160 | 2.83E-54 | 683 | 7.28 |
| N5 | 820 | AwTGGGCy | 259 | 6146 | 2.16E-20 | 738 | 15.86 |
| N8 | 88 | kACGTGkn | 48 | 7194 | 1.16E-11 | 710 | 5.65 |
| N9 | 96 | rCCGACry | 48 | 2094 | 3.12E-32 | 672 | 4.58 |
| N10 | 355 | ACGTGkmn | 166 | 8607 | 7.98E-18 | 638 | 7.23 |
| N11 | 295 | mCGCGTnn | 82 | 3760 | 3.89E-15 | 654 | 5.14 |
| N12 | 197 | mCGCGTnn | 87 | 3760 | 8.59E-32 | 709 | 7.95 |
| N13 | 150 | GmCACGTn | 76 | 4849 | 1.13E-25 | 727 | 7.95 |
| N14 | 302 | AGGGTTTw | 152 | 5787 | 8.26E-40 | 870 | 18.41 |
| N15 | 205 | TGTmTATr | 105 | 9728 | 2.31E-11 | 598 | 3.77 |
| N17 | 168 | sCGrTTss | 49 | 4321 | 2.14E-08 | 597 | 2.35 |
| N18 | 465 | nGGCCCAn | 304 | 8287 | 2.14E-77 | 854 | 26.74 |
| N19 | 217 | TTGACTTy | 101 | 5626 | 1.63E-24 | 624 | 5.06 |
| N20 | 96 | rACACGwr | 53 | 6152 | 6.41E-16 | 591 | 2.5 |
| N21 | 79 | kACGTGkm | 37 | 4363 | 2.49E-13 | 813 | 7.43 |
