# Supplementary material for: Discovery of Stress Responsive DNA Regulatory Motifs in Arabidopsis
Source: PLoS One. 2012 Aug 13;7(8):e43198. doi: 10.1371/journal.pone.0043198 (PMC3418279; doi:10.1371/journal.pone.0043198)
Supplement: Table S2 — Major 8-mer motifs identified for coexpressed genes from various clusters. (DOC) [file pone.0043198.s002.doc]

Table S2. Major 8-mer motifs identified for coexpressed genes from various clusters

| **Cluster** | **Cluster Size** | **Motif** | **In cluster** | **In genome** | **pValue** | **Mean position** | **z score for TSS factor** | **Similar to motif** | **Motif sequences** |
| --- | --- | --- | --- | --- | --- | --- | --- | --- | --- |
| **Genes induced by various biotic and abiotic stresses** | | | | | | | | | |
| N11 | 300 | mCGCGTnn | 82 | 3760 | 3.89E-15 | 654 | 5.14 | CGCGBOXAT | vCGCGb |
|  | 300 | rGTCAAAs | 91 | 5462 | 5.25E-10 | 595 | 3.39 | WBBOXPCWRKY1 | TTTGACy |
|  | 300 | GACTTTkn | 152 | 11911 | 2.03E-08 | 607 | 5.19 |  |  |
|  | 300 | CGTGTkwn | 120 | 8796 | 7.87E-08 | 597 | 4.04 |  |  |
|  | 300 | TATAwAGn | 193 | 16392 | 1.07E-08 | 563 | 3.47 |  |  |
| **Genes induced by plant stress hormone abscisic acid (ABA)** | | | | | | | | | |
| N3 | 154 | GmCACGTr | 92 | 3160 | 2.83E-54 | 683 | 7.28 | ABREATCONSENSUS | yACGTGGC |
|  | 154 | **CACGCGyn** | 35 | 1683 | 3.79E-14 | 641 | 3.01 | n/a |  |
|  | 154 | TsACGTGn | 52 | 4294 | 1.60E-11 | 607 | 2.79 |  |  |
|  | 154 | **kAACGTrn** | 68 | 7333 | 8.31E-10 | 523 | 0.67 | n/a |  |
|  | 154 | GTCGGynr | 49 | 4226 | 3.65E-10 | 588 | 2.18 | DRECRTCOREAT | rCCGAC |
|  | 154 | **GGACGryn** | 38 | 3969 | 8.29E-06 | 686 | 3.99 | n/a |  |
| N9 | 96 | rCCGACry | 48 | 2094 | 3.12E-32 | 672 | 4.58 | DRECRTCOREAT | rCCGAC |
|  | 96 | ACGTGkCn | 43 | 4848 | 8.60E-13 | 674 | 4.71 | ACGTABREMOTIFA2OSEM | ACGTGkC |
|  | 96 | rGTCGGym | 21 | 1645 | 6.47E-09 | 700 | 3.3 | Agris_DRE-like promoter motif | drCCGACnw |
|  | 96 | ATGTCGAy | 18 | 1374 | 6.29E-08 | 659 | 2.31 | CRTDREHVCBF2 | GTCGAC |
|  | 96 | mACrCGkn | 53 | 8931 | 3.86E-09 | 627 | 3.7 | QARBNEXTA | AACGTGT |
| N10 | 355 | ACGTGkmn | 166 | 8607 | 7.98E-18 | 638 | 7.23 | ACGTABREMOTIFA2OSEM | ACGTGkC |
|  | 355 | **AAAArAGT** | 167 | 11022 | 2.89E-08 | 540 | 1.93 | n/a |  |
|  | 355 | mACrCGnn | 212 | 15266 | 9.41E-08 | 600 | 6.16 | CGCGBOXAT | vCGCGb |
|  | 355 | TTwCGTAm | 70 | 3670 | 1.24E-06 | 608 | 3.14 | ACGTABOX | TACGTA |
|  | 355 | AmTTAAAA | 193 | 13912 | 9.86E-07 | 557 | 3.18 | POLASIG2 | AATTAAA |
| N13 | 150 | GmCACGTn | 76 | 4849 | 1.13E-25 | 727 | 7.95 | ACGTABREMOTIFA2OSEM | ACGTGkC |
|  | 150 | nCGTGTrn | 85 | 11106 | 4.60E-09 | 587 | 3.15 | QARBNEXTA | AACGTGT |

(Putative new motifs are marked bold)
