# Supplementary material for: Discovery of Stress Responsive DNA Regulatory Motifs in Arabidopsis
Source: PLoS One. 2012 Aug 13;7(8):e43198. doi: 10.1371/journal.pone.0043198 (PMC3418279; doi:10.1371/journal.pone.0043198)
Supplement: Table S3 — Major 12-mer motifs over-represented in stress responsive genes promoters. (DOC) [file pone.0043198.s003.doc]

Table S3. Major 12-mer motifs over-represented in stress responsive genes promoters

| **Cluster** | **Cluster Size** | **Motif** | **In cluster** | **In genome** | **pValue** | **Mean position** | **z score for TSS factor** |
| --- | --- | --- | --- | --- | --- | --- | --- |
| N18 | 465 | nnTGGGCCnnnn | 304 | 8272 | 1.34E-77 | 855 | 29.25 |
|  | 465 | nnAAGCCCAnnn | 198 | 5812 | 1.22E-37 | 815 | 19.52 |
|  | 465 | nnAAACCCTAnn | 165 | 4981 | 1.17E-28 | 808 | 16.4 |
| N0 | 712 | TTGACTTnnnnn | 333 | 8754 | 5.56E-33 | 590 | 6.27 |
|  | 712 | GTCAACnnnnnn | 355 | 10887 | 3.20E-22 | 587 | 6.62 |
|  | 712 | GTCAAAnAnnnn | 280 | 8453 | 6.06E-17 | 618 | 7.58 |
|  | 712 | AnCGCGTnnnnn | 136 | 3077 | 1.65E-16 | 671 | 7.24 |
|  | 712 | AnGACTTTTnnn | 121 | 2676 | 2.10E-15 | 615 | 4.57 |
|  | 712 | AnTAnTAnnnAA | 451 | 16729 | 3.46E-13 | 530 | 2.57 |
|  | 712 | TAnTTnnTAAnn | 438 | 16150 | 5.88E-13 | 520 | 1.58 |
|  | 712 | TCAAAnTnAnnn | 412 | 14947 | 7.83E-13 | 503 | 0 |
|  | 712 | nTnGAATnTTnn | 360 | 12617 | 1.49E-12 | 540 | 2.85 |
| N12 | 197 | AnCGCGTnnnnn | 74 | 3077 | 2.18E-27 | 708 | 7.75 |
|  | 197 | nACGTGTnnnnn | 110 | 9027 | 1.23E-17 | 603 | 4.88 |
|  | 197 | ATAATTTnGTnn | 38 | 1955 | 6.44E-11 | 466 | -0.95 |
|  | 197 | ACACGCnnnnnC | 26 | 1014 | 3.37E-10 | 675 | 3.44 |
| N3 | 154 | nGnnACGTGnnn | 107 | 6664 | 1.63E-40 | 676 | 8.59 |
|  | 154 | CnnACACGTnnA | 27 | 680 | 9.99E-18 | 613 | 2.27 |
|  | 154 | CACGCGnnnnnn | 40 | 2611 | 7.31E-12 | 631 | 3.29 |
| N7 | 82 | CGTGnGnGGCAC | 7 | 21 | 4.56E-14 | 904 | 3.71 |
|  | 82 | CAACnTAGCnCG | 5 | 18 | 6.47E-10 | 726 | 1.74 |
