# Supplementary material for: Discovery of Stress Responsive DNA Regulatory Motifs in Arabidopsis
Source: PLoS One. 2012 Aug 13;7(8):e43198. doi: 10.1371/journal.pone.0043198 (PMC3418279; doi:10.1371/journal.pone.0043198)
Supplement: Table S4 — Major motifs identified from Arabidopsis genes induced by Pseudomonas syringae pv. tomato ( Pst ) (avrRpm1) – a case study on a single microarray experiment. (DOC) [file pone.0043198.s004.doc]

Table S4. Major motifs identified from Arabidopsis genes induced by

*Pseudomonas syringae* pv. tomato (*Pst*) (avrRpm1) – a case study on

a single microarray experiment

| **Motif** | **Cluster Size** | **In Cluster** | **In Genome** | **pValue** | **Mean Position** | **Z score for TSS** |
| --- | --- | --- | --- | --- | --- | --- |
| rCGTGTnn | 461 | 292 | 11958 | 6.98E-34 | 618 | 8.86 |
| ATATTwTA | 461 | 258 | 12964 | 4.77E-14 | 509 | 0.42 |
| TCTAGAmr | 461 | 110 | 3870 | 1.07E-13 | 591 | 3.44 |
| rCGTAwny | 461 | 246 | 12433 | 1.20E-12 | 542 | 2.55 |
| TTTGACnw | 461 | 280 | 14964 | 3.77E-12 | 578 | 5.29 |
| wrTTGACn | 461 | 284 | 15283 | 4.26E-12 | 525 | 1.53 |
| TAnATACw | 461 | 298 | 16375 | 7.20E-12 | 561 | 4.22 |
| yATTCAAm | 461 | 206 | 10024 | 1.83E-11 | 543 | 2.25 |
| GAAnmkTC | 461 | 241 | 12681 | 2.49E-10 | 556 | 3.45 |
| mCrCGGnn | 461 | 163 | 8401 | 7.49E-07 | 566 | 3.11 |
| ACGArAAn | 461 | 198 | 10895 | 2.34E-06 | 567 | 3.49 |
