# Supplementary material for: Discovery of Stress Responsive DNA Regulatory Motifs in Arabidopsis
Source: PLoS One. 2012 Aug 13;7(8):e43198. doi: 10.1371/journal.pone.0043198 (PMC3418279; doi:10.1371/journal.pone.0043198)
Supplement: Table S5 — Motifs with position bias only in Arabidopsis. (DOC) [file pone.0043198.s005.doc]

Table S5. Motifs with position bias only in Arabidopsis

|  |  |  | **Arabidopsis** | | | **Rice** | | |
| --- | --- | --- | --- | --- | --- | --- | --- | --- |
| **Motif** | **Similar to Known Motif** | **Known Motif Sequence** | **Instances** | **Mean position** | **z-score** | **Instances** | **Mean position** | **z-score** |
| TwTTTATw | Place_SEF1MOTIF | ATATTTAww | 29597 | 553 | 33.413 | 24763 | 495 | -0.94 |
| AAAAAwAs | n/a |  | 28421 | 550 | 31.038 | 30046 | 500 | 1.98 |
| TArACCGr | n/a |  | 2885 | 629 | 24.724 | 1376 | 518 | 2.728 |
| CCnATTAn | Place_WUSATAg | TTAATGG | 9520 | 561 | 21.85 | 8533 | 504 | 2.342 |
| AAACGwCr | Place_BOXBPSAS1 | AAACGACACCGTTT | 3033 | 604 | 20.47 | 1964 | 485 | -1.926 |
| CCyAATAw | n/a |  | 3680 | 590 | 19.577 | 3252 | 506 | 1.728 |
| CCnAnTTA | Agris_SORLREP4 | CTCCTAATT | 10466 | 551 | 19.436 | 12214 | 502 | 1.895 |
| GnCnTTTA | n/a |  | 9702 | 552 | 19.007 | 7195 | 503 | 1.747 |
| GGnCCTTA | n/a |  | 1196 | 645 | 17.866 | 1503 | 512 | 1.96 |
| TrAGCCyA | n/a |  | 2125 | 607 | 17.608 | 3175 | 505 | 1.628 |
| GGnTTwAA | n/a |  | 8203 | 551 | 17.184 | 6924 | 504 | 1.917 |
| CCGGTTCr | n/a |  | 898 | 649 | 15.845 | 860 | 514 | 1.778 |
| GGGnCTAA | n/a |  | 1328 | 619 | 15.561 | 2402 | 507 | 1.662 |
| yCGGTTAw | Place_GT1CORE | GGTTAA | 2119 | 592 | 15.211 | 1264 | 511 | 1.784 |
| yACGTGTw | Agris_Z-box promoter motif | ATACGTGT | 2285 | 588 | 15.155 | 2622 | 513 | 2.783 |
| TATArAGr | n/a |  | 5175 | 556 | 14.911 | 3790 | 509 | 2.679 |
| AGGGTnwT | Place_UP2ATMSD | AAACCCTA | 4082 | 562 | 14.52 | 4574 | 508 | 2.508 |
| TwATTTCs | n/a |  | 4770 | 555 | 13.969 | 3654 | 494 | -0.592 |
| TAAArCGn | Place_SORLREP2AT | ATAAAACGT | 4395 | 555 | 13.417 | 3993 | 508 | 2.325 |
| AnTAwGGG | n/a |  | 3129 | 563 | 12.92 | 4322 | 488 | -1.988 |
| AwGACACG | Place_ABREMOTIFAOSOSEM | TACGTGTC | 909 | 617 | 12.659 | 985 | 524 | 2.973 |
| GGsTAwAA | n/a |  | 2377 | 571 | 12.644 | 3686 | 505 | 1.654 |
| ArrGTCAA | Place_WBBOXPCWRKY1 | TTTGACy | 4410 | 550 | 12.202 | 3983 | 478 | -4.099 |
| ATnACGTs | Place_PALINDROMICCBOXGM | TGACGTCA | 2970 | 560 | 11.95 | 3327 | 510 | 2.703 |
| ATAGrCyC | n/a |  | 1307 | 592 | 11.926 | 1674 | 505 | 1.107 |
| TTAATTAA | Place_POLASIG2 | AATTAAA | 3029 | 556 | 11.288 | 3232 | 496 | -0.25 |
| CCsTTAnA | n/a |  | 2673 | 557 | 10.898 | 3497 | 503 | 1.151 |
