# Supplementary material for: Discovery of Stress Responsive DNA Regulatory Motifs in Arabidopsis
Source: PLoS One. 2012 Aug 13;7(8):e43198. doi: 10.1371/journal.pone.0043198 (PMC3418279; doi:10.1371/journal.pone.0043198)
Supplement: Table S6 — Motifs with position bias only in Rice. (DOC) [file pone.0043198.s006.doc]

Table S6. Motifs with position bias only in Rice

|  |  |  | **Arabidopsis** | | | **Rice** | | |
| --- | --- | --- | --- | --- | --- | --- | --- | --- |
| **Motif** | **Similar to Known Motif** | **Known Motif Sequence** | **Instances** | **Mean position** | **z-score** | **Instances** | **Mean position** | **z-score** |
| CCCnCCnC | Agris_E2F-varient binding site | TCTCCCGCC | 1689 | 507 | 1.495 | 21467 | 588 | 46.359 |
| GGnTGGGn | n/a |  | 2561 | 506 | 1.507 | 14491 | 604 | 44.893 |
| GGsnCCAC | n/a |  | 899 | 525 | 2.934 | 7749 | 631 | 40.981 |
| CTCnnCTC | n/a |  | 7374 | 498 | 0.341 | 22662 | 572 | 39.423 |
| CGCGnGsG | n/a |  | 258 | 532 | 1.952 | 7304 | 600 | 30.708 |
| GAGnnGGA | n/a |  | 6709 | 479 | -5.229 | 21551 | 551 | 27.497 |
| CCCAnwCC | n/a |  | 1904 | 507 | 1.541 | 5624 | 602 | 27.375 |
| GGnTTTGn | Place_UP2ATMSD | AAACCCTA | 14975 | 503 | 2.583 | 17094 | 555 | 26.289 |
| CGCGnnGC | Place_ABREMOTIFIIIOSRAB16B | GCCGCGTGGC | 642 | 505 | 0.682 | 11575 | 566 | 25.884 |
| CAsnTGGG | Agris_CBF2 binding site | CCACGTGG | 1442 | 504 | 0.989 | 5822 | 592 | 25.348 |
| CCCnTnTC | n/a |  | 4157 | 508 | 2.533 | 12898 | 560 | 24.811 |
| GGnCnGGA | n/a |  | 1589 | 507 | 1.364 | 7713 | 577 | 24.472 |
| CCACACnn | Place_5256BOXLELAT5256 | TGTGGTTATATA | 4983 | 504 | 1.838 | 11608 | 561 | 24.214 |
| ACnGCnCA | n/a |  | 2641 | 511 | 2.453 | 8797 | 570 | 23.848 |
| CCCACGrn | n/a |  | 908 | 509 | 1.251 | 3904 | 606 | 23.731 |
| CnCGAnCC | n/a |  | 2357 | 503 | 1.024 | 6959 | 578 | 23.479 |
| GGAAnGGn | Place_AMMORESIIUDCRNIA1 | GGwAGGGT | 3823 | 480 | -3.576 | 11747 | 556 | 22.14 |
| ATysGACG | n/a |  | 864 | 524 | 2.762 | 2364 | 625 | 21.659 |
| CnTCTTCy | Place_TCA1MOTIF | TCATCTTCTT | 12736 | 503 | 2.428 | 12765 | 550 | 20.754 |
| ACCrnCCC | Place_ACIIPVPAL2 | CCACCAACCCCC | 1272 | 518 | 2.627 | 4435 | 585 | 20.348 |
| AGCGAGns | n/a |  | 1149 | 510 | 1.507 | 4474 | 584 | 20.178 |
| CTGACnnG | Agris_TGA1 binding site | TGACGTGG | 2671 | 511 | 2.441 | 9039 | 556 | 19.52 |
| GCCnGnCC | n/a |  | 847 | 525 | 2.866 | 7626 | 560 | 19.173 |
| GGrwGGAA | n/a |  | 1518 | 464 | -4.48 | 4736 | 575 | 18.687 |
| GCGGCyyA | n/a |  | 325 | 521 | 1.5 | 1919 | 619 | 18.653 |
| CCCAnCrA | n/a |  | 2687 | 506 | 1.588 | 5528 | 569 | 18.649 |
| ATCCAnCs | Place_NONAMERMOTIFTAH3H4 | CATCCAACG | 2757 | 510 | 2.448 | 7104 | 560 | 18.426 |
| CCCwnGCC | n/a |  | 770 | 511 | 1.354 | 5659 | 566 | 18.114 |
| CnCGnATC | Agris_octamer promoter | CGCGGATC | 2709 | 495 | -0.409 | 5827 | 565 | 18.109 |
| GGnCnAGA | n/a |  | 4023 | 504 | 1.624 | 7679 | 554 | 17.326 |
| GCTyTCnC | n/a |  | 3165 | 487 | -2.043 | 5775 | 562 | 17.235 |
| GAGGCnyA | n/a |  | 1539 | 515 | 2.449 | 3931 | 576 | 17.229 |
| AGsnAAGC | n/a |  | 2504 | 479 | -3.054 | 5710 | 562 | 17.038 |
| CGCCTwTn | n/a |  | 1196 | 520 | 2.779 | 2468 | 592 | 16.488 |
| ACGCAnnC | Place_GCAACREPEATZMZEIN | GCAACGCAAC | 2267 | 495 | -0.308 | 7055 | 553 | 16.436 |
| GGsGnTTA | n/a |  | 1463 | 519 | 2.939 | 2696 | 583 | 15.537 |
| GGGnTTCn | n/a |  | 4099 | 508 | 2.529 | 6737 | 551 | 15.456 |
| GnGAGTsA | Agris_SORLIP5 | GAGTGAG | 3021 | 510 | 2.519 | 4224 | 564 | 15.098 |
| CGTTTCsn | n/a |  | 2362 | 515 | 2.993 | 3641 | 568 | 14.832 |
| CCCGnAAn |  |  | 2393 | 486 | -1.815 | 4151 | 563 | 14.818 |
| GrGCnGAA | n/a |  | 1813 | 493 | -0.592 | 4316 | 560 | 14.489 |
| ACyACyAC | n/a |  | 2246 | 483 | -2.277 | 5616 | 552 | 14.423 |
| CsCAACrC | Place_GCAACREPEATZMZEIN | GCAACGCAAC | 649 | 490 | -0.635 | 1975 | 589 | 14.324 |
| CGrTCGnG | n/a |  | 665 | 498 | 0.122 | 3391 | 567 | 14.234 |
| CCCGCAsn | Place_TE2F2NTPCNA | ATTCCCGC | 342 | 433 | -4.136 | 3004 | 570 | 13.947 |
| CACrTCnC | Place_ABREBZMRAB28 | TCCACGTCTC | 2373 | 513 | 2.649 | 5662 | 550 | 13.905 |
| CCCAnCTw | Place_ACIPVPAL2 | CCCACCTACC | 2617 | 512 | 2.643 | 5633 | 550 | 13.78 |
| GGTCnCTs | n/a |  | 1461 | 498 | 0.081 | 3587 | 562 | 13.671 |
| GAGwCGCn | n/a |  | 1010 | 487 | -1.065 | 2608 | 573 | 13.501 |
| AAnCwCCC | n/a |  | 2159 | 498 | 0.19 | 5089 | 550 | 13.29 |
| CGwGTCCn | Place_ABRETAEM | GGACACGTGGC | 1191 | 521 | 2.89 | 3354 | 562 | 13.151 |
| CGCsATTn | n/a |  | 1687 | 508 | 1.585 | 3158 | 564 | 13.104 |
| CnGCGAAw | Place_E2FCONSENSUS | wTTssCss | 1555 | 500 | 0.471 | 3217 | 562 | 12.76 |
| GCCwGCCw | n/a |  | 366 | 497 | -0.01 | 2866 | 565 | 12.674 |
| CCCAnwGG | n/a |  | 1343 | 518 | 2.657 | 2888 | 562 | 12.193 |
| GAwGCGAw | n/a |  | 1075 | 472 | -2.896 | 1905 | 577 | 12.152 |
| AAGCGGrn | Place_BS1EGCCR | AGCGGG | 1130 | 472 | -2.896 | 2890 | 562 | 12.145 |
| CnCCTrTA | n/a |  | 1581 | 516 | 2.585 | 4131 | 551 | 12.022 |
| CCCAnCrG | n/a |  | 1065 | 473 | -2.674 | 3991 | 551 | 11.895 |
| CGCTwTAw | n/a |  | 989 | 515 | 1.984 | 1465 | 585 | 11.688 |
| GsCCATGr | n/a |  | 920 | 522 | 2.673 | 3322 | 555 | 11.672 |
| TCTnGGGs | n/a |  | 957 | 497 | 0.032 | 2670 | 562 | 11.625 |
| CTTwTTCC | n/a |  | 2073 | 516 | 2.976 | 3304 | 554 | 11.516 |
| AATCnGsG | n/a |  | 1273 | 488 | -1.157 | 2650 | 559 | 11.085 |
| CCCrAsTC | n/a |  | 839 | 499 | 0.206 | 2097 | 565 | 10.871 |
| CsGTnCAC | n/a |  | 1037 | 520 | 2.558 | 3372 | 551 | 10.863 |
| AACynGGC | n/a |  | 1398 | 510 | 1.643 | 3472 | 550 | 10.846 |
| AwAyACGC | n/a |  | 1028 | 509 | 1.305 | 1880 | 568 | 10.775 |
| sCCCGGGn | Place_AMMORESVDCRNIA1 | GGCCCCGGG | 147 | 467 | -1.249 | 1551 | 574 | 10.635 |
| GAGACGry | n/a |  | 1326 | 501 | 0.466 | 2608 | 556 | 10.501 |
